# How does ageism influence frailty? A preliminary study using a structural equation model

**DOI:** 10.1186/s12877-020-01749-8

**Published:** 2020-10-26

**Authors:** Bo Ye, Junling Gao, Hua Fu, Hao Chen, Wenjing Dong, Ming Gu

**Affiliations:** 1 School of Public Health, Fudan University, PO Box 248, 138 Yixueyuan Road, Shanghai, 200032 China; 2 Fudan Health Communication Institute, School of Public Health, Fudan University, PO Box 248, 138 Yixueyuan Road, Shanghai, 200032 China

**Keywords:** Ageism, Frailty, Experiences of ageism, Age stereotypes, Attitudes to ageing

## Abstract

**Background:**

Based on the Stereotype Embodiment Theory (SET), this study aims to examine the mechanism of ageism on frailty through the proposed framework of “Experiences of Ageism (EA) → Age Stereotypes (AS) → Attitudes to Ageing (AA) → Frailty” using a structural equation model (SEM).

**Methods:**

A community-based study involving 630 participants aged 60 years and older was conducted in Shanghai. EA, AS, AA and frailty status were assessed by validated scales. In particular, EA included three parts in this study, as the first part was the experiences of explicit prejudice or discrimination because of age, another two parts were the experiences of witnessed and encountered implicit negative age-based stereotypes. A SEM was performed to examine whether the proposed paths from EA to frailty were supported.

**Results:**

EA had a significant indirect effect (β’ = .360*-.456*-.576 = .095, *p* < .001) on frailty through the path of “EA → AS → AA → Frailty” after controlling for covariates. AA had a direct effect (β = −.576, *p* < .001) on frailty; AS fully mediated the association between EA and AA (indirect effect = .360*-.456 = −.164, *p* < .001), and AA fully mediated the association between AS and frailty (indirect effect = −.456*-.576 = .263, *p* < .001).

**Conclusions:**

These findings demonstrated a mechanism from ageism to frailty, and highlighted the potential threat of negative AS on health. Ageism and frailty are both great challenges for the process of healthy ageing.

## Background

Frailty is defined as a progressive age-related deterioration in physical systems that leads to extreme vulnerability to stressors and increases the risk of many adverse health outcomes or even death [1–3]. It is regarded as a modern geriatric giant and a major public health problem in the ageing population [3]. Frailty has been proved to be influenced by various of factors, which mostly in physical aspect; however, psychological factors may also play important roles in this process. Currently, a longitudinal study showed that older adults’ attitudes to ageing had a significant prediction on physical frailty status [4]. Importantly, it will increase the perceptions of older people as a burden, which may lead to a higher risk of ageism in current quick ageing world [5]. Older people who perceived ageism may have direct negative effects on their health and well-being [6–8].

Ageing process is widely assumed as an entirely physiological process of inevitable decline. Some of age-based stereotypes and prejudices arise from observable biological declines, and this may distort the perceptions about older people and general ageing process (a typical example is dementia, which may be mistakenly thought to reflect normal ageing) [9]. Based-on so-called facts and presumed perceptions, the health of old people is often threatened by these stereotypes intentionally or unintentionally [10, 11]. On the other hand, older adults can be seen as warm but incompetent, which leads to paternalistic prejudice and patronizing behaviour [12, 13]. These may seem benign, but are associated with negative outcomes [14, 15].

The stereotype embodiment theory (SET) proposed that individuals who are more frequently exposed to stereotypes are more likely to embody such stereotypes [16]. Experiences of AS (also include ageism) probably enhance such negative AS and embody them (negative attitudes to ageing), all these age-based factors that older adults experienced or hold can directly and/or indirectly influence their physical health. However, rarely studies clarified the mechanism from experiences of AS and ageism to frailty among older adults. Therefore, this study provides a preliminary examination and explanation about this pathway.

### Experiences of ageism, age stereotypes and attitudes to ageing

Ageism is widely defined as the stereotyping, prejudice and discrimination towards people based on age [17]. Although old age was honoured and respected in a more traditional societies [18], attitudes and stereotypes about older people focus predominantly on the negative aspects of ageing, and older age typecast as an inevitable decline in physical and mental capacities leading to frail, burdensome or dependent were ubiquitous perceptions [19, 20]. Older people were often the primary targets/victims [16, 21], and became increasingly vulnerable to the effects of ageism with growing older [22]. Many previous studies have examined that perceived ageism had direct influence on older adults’ health outcomes, such as self-rated health [8, 23, 24], depressive symptoms [6, 25]. According to a broader concept of ageism, experiences of ageism (EA) not only refers to older people who perceive that they have experienced prejudice or discrimination as results of their age, but also situations based on AS that old people have witnessed and/or encountered.

Ageism also comes from the perception that an elder might be too old to be or to do something [20]. Older adults may be clearly aware of being regarded as ‘old’, but are often uncertain to protest that they are actively treated as elder or discriminated against because of their age [26]. Because these age-based stereotypes and perceptions are unconsciously internalizing across the life-course [16, 20], and the implicit ageist assumptions and ideas in our life and culture are often presented in daily interactions [26]. For instance, an old people might be told “you are too old to do that, you are more likely to get hurt”; and another example, when an older person forgot something, he/she usually blurted out “I am old”. Incompetence (physical domain) and memory loss (cognitive domain) are often referred to as the ageing process, which represented the most focused aspects of AS [27–30]. Older adults may be restricted by the excuse of age and also may attribute their incompetence to age themselves. EA can be from others, but also from themselves [20], both can have long-term effects on older people’s health.

Previous studies showed that, for the old people, experiences of age-related changes seemed to influence their AS [31, 32], which refers to general beliefs about older adults [16, 31]. People tend to seen their experiences as normal and thus influence their general attitudes toward the in-group they belong to, this is described as the process of stereotype projection [33]. In other words, older adults’ negative AS probably be enhanced by age-related experiences, which include witnessing or encountering instances of age-based stereotyping, prejudice, and discrimination. Individuals are more likely to integrate stereotypical information into their ideas of ageing when confront with these age-related experiences [34, 35]. AS was proved to have direct effects on older adults’ physiologic stress response [36], and many other previous studies have showed that the activation of AS can influence older adults’ physical functioning performance [10, 27, 37, 38]. Furthermore, AS was showed direct effects on older adults’ gait speed [37], which is one of the core components of frailty [1].

As mentioned above, attitudes to ageing (AA) has been presented a predictor of frailty. It reflects older adults’ beliefs about both their own ageing and general ageing in physical, psychological and psychosocial domains [39]. The SET explains that the ageing process is related to a social construct, and AS is usually embodied through multiple pathways. Individuals internalize the AS across the life span while assimilating perceptions with their culture, and this AS can eventually result in beneficial or detrimental effects on elders’ functioning and health [16]. According to this theory, older adults who were more frequently exposed to AS and/or ageism (EA) are more likely to enhance and embody such AS, which probably manifest in their AA, and then influence health. Previous studies have examined that AA (or self-perception of ageing (SPA), which means older individuals’ beliefs about their own ageing) has direct effects on health-related outcomes, such as subjective health [40], objective functioning [41], and even mortality [42]. The effects of SPA on physical functioning, rather than physical functioning impacting SPA, were also demonstrated in a previous study [43]. Most of studies about AA focused on its health effects, rather than its influence factors. However, a few of studies still provide the evidences that EA has a negative effect on mental health through the mediator of SPA [44, 45], and AS has a significant effect on physical functioning through the mediator of SPA [10].

**Fig. 1:**
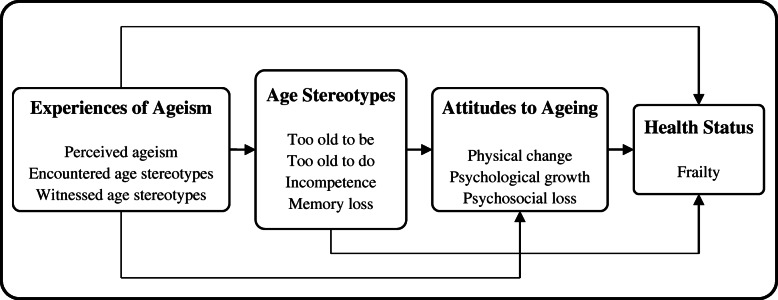
Hypothesized model of “EA → AS → AA → Frailty” pathway

Therefore, based on existing theory and evidences, we put forward a hypothesized model of “EA → AS → AA → Frailty” pathway in Fig. 1. The current study extends previous research by examining the effects of ageism and its psychological pathway on frailty for older adults using a structural equation model (SEM).

## Methods

### Participants and procedure

Six hundred and thirty Chinese older adults (≥60 years) were surveyed in Shanghai during January 2019. A dozen of investigators with unified training completed this survey using iPads or paper questionnaires. These targeting older Chinese were reached in several communities using a diverse range of recruitment strategies, which included family doctors’ advice to their older targets, community workers’ introduction in the neighbourhood centre, investigators’ initiative recruitment outdoor within neighbourhoods and encouraging referrals from participants themselves. For instance, we surveyed parts of the autonomous participants under the help of family doctors and community workers; another part of participants was from snowball sampling; and some other participants were completed through the measure of household survey with calls ahead. Participation was voluntary, and participants were informed that their responses were anonymous and confidential before starting the survey. It took participants about 30 min to complete the survey and older people with severe mental or cognitive disorder were excluded. Finally, there was no missing data under a strict quality control, and of these all 630 participants comprised the final sample for statistical analysis. The current group ranged in age from 60 to 94 years, with a mean age of 74.19 years (Standard Deviation (SD) = 8.53). Table 1 shows descriptive statistics for sociodemographic variables, attitudes to ageing and frailty status.

**Table 1:** Descriptive statistics for sociodemographic variables, attitudes to ageing and frailty status

Characteristics	Total (%)	Characteristics	Total (%)
Gender		Economic condition	
Male	228 (36.2)	Income lower than expenditure	75 (11.9)
Female	402 (63.8)	Income equal expenditure	339 (53.8)
Age group		Income higher than expenditure	216 (34.3)
60–69 years	219 (34.8)	Residence status	
70–79 years	205 (32.5)	Live alone	123 (19.5)
≥ 80 years	206 (32.7)	Live with spouse	337 (53.5)
Education		Live with others	170 (27.0)
Illiteracy	51 (8.1)	Attitude to ageing (Mean ± SD)	
Primary school	75 (11.9)	Psychological growth (8–40)	26.82 ± 4.02
Junior high school	229 (36.3)	Physical change (8–40)	27.69 ± 4.76
High school or equivalent	179 (28.4)	Psychosocial loss (8–40)	20.61 ± 5.19
College or above	96 (15.3)	Frailty status	
Marital status		Robust	272 (43.2)
Married	458 (72.7)	Prefrail	263 (41.7)
Unmarried	172 (27.3)	Frail	95 (15.1)

### Assessments and measures

**Experiences of ageism (EA)** was measured with 11 questions including three aspects: 1) perceived ageism; 2) encountered AS; and 3) witnessed AS. Perceived ageism was measured by three questions: “*How often, in the last year, has anyone shown prejudice against you or treated you unfairly because of your age?*”; “*How often, if at all, in the last year have you felt that someone showed you a lack of respect because of your age, for instance by ignoring or patronizing you?*”; and “*How often in the last year has someone treated you badly because of your age, for example by insulting you, abusing you or refusing you services?*” All response scales ranged from 0 *never* to 4 *very often* [24]. Encountered AS and witnessed AS were both consist of 4 similar questions, which were derived from the general perceptions (“*An elder might be too old to be or to do something*”) [20] and stereotypes (such as *incompetence* and *memory loss*) [27–30] based on the older age. For example, we ask the participants: “*How often, in the past year, has anyone told you ‘as an older people, you should be…rather than…’?*” and “*How often, in the past year, have you witnessed someone told an old person ‘as an older people, you should be…rather than…’?*”; “*How often, in the past year, has anyone told you ‘you are too old for that, it’s for young’?*” and “*How often, in the past year, have you witnessed someone told an old person ‘you are too old for that, it’s for young’?*”; “*How often in the past year have you encountered someone doubted your competence because of your older age? such as don’t believe you understand well or you are more likely make mistakes if comparing to young*” and “*How often, in the past year, have you witnessed someone doubted an old person’s competence because of his/er older age?*”; and “*How often, in the past year, has anyone told you that’s so-called a ‘senior moment’ when you forgot something?*” and “*How often, in the past year, have you witnessed anyone blurt out ‘I’m old or muddled or useless’ when s/he cannot remember something?*”. All response scales also ranged from 0 *never* to 4 *very often*. Given the extremely skewed distribution of the responses to these measures, we recoded each item as a dichotomy with consulting previous study [23, 24]. Older people who scored 1 or above on each item indicated a positive result, which was regarded as having experiences of ageism. For testing the psychometrics of this EA scale, we performed exploratory factor analysis (EFA) and confirmatory factor analysis (CFA) with method of Maximum Likelihood (ML) to examine the structure validity. The initial eigenvalues (number > 1) from the EFA showed a model with 3 components, which exactly fitted the proposed aspects and explaining 73.6% of the total variance. All of the three varimax-rotated components showed over 20% of the total variance (22.3, 22.0 and 20.1%, respectively), and we also inspected for items that had acceptable loadings (>.50) (see Additional file 1). The modified indices of the CFA were as follows: χ^2^ = 108.049, df = 38 (χ^2^/df = 2.843); and the requisite fitness parameters were within acceptable standards (Comparative Fit Index (CFI) = .982, Tucker-Lewis Index (TLI) = .975, Root Mean Square Error of Approximation (RMSEA) = .054) (also see Table 3). The Cronbach’s alpha for the three components of EA scale were .884, .858 and .857 respectively in the current study.

**Age stereotypes (AS)** is usually measured by rating “old people” on some personal characteristics or domains in their lives [31]. In current study, AS was assessed with the similar set of statements, which were used for measurement of encountered or witnessed AS in the four aspects. Participants had to rate “dis/agree” instead of rating “frequency”. We asked participants: “*What extent do you disagree or agree with the following statements*: ‘*as an older people, it should be…rather than…*’; ‘*older people are too old for something, it’s for young*’; ‘*comparing to young, older people are more likely to make mistakes*’; ‘*the older a person is, the more likely to be forgetful or muddled*’”. These four response scales ranged from 1 *strongly disagree* to 5 *strongly agree*. The initial eigenvalues (number > 1) from the EFA suggested a model with 1 component, explaining 67.3% of the total variance, and the varimax-rotated component was also inspected for items that had acceptable loadings (>.50) (also see Additional file 1). The modified indices of the CFA for the AS were also adequate (see Table 3). The Cronbach’s alpha for the AS scale was .832 in the current study.

**Attitudes to ageing (AA)** was measured with the attitudes to ageing questionnaire (AAQ), which was developed and validated by Laidlaw K, et al. in a worldwide cross-culture populations [39]. This questionnaire has been validated in multiple culture, also including a Chinese version [46]. The 24-item questionnaire was evenly divided into three domains including psychological growth (PG), physical change (PC) and psychosocial loss (PL) with acceptable Cronbach’s alpha of .592, .760 and .790, respectively. The questionnaire uses a Likert response format for each item from 1 *strongly disagree* to 5 *strongly agree*. PG focuses on the wisdom and growth, which reflects both positive gains in relation to self and to others about ageing; PC emphasizes the positive beliefs on maintaining physical health and the experience of ageing itself; PL presents negative experiences involving psychological and social loss in old age [39]. Higher summated scores in each dimension for PC and PG indicate a more positive perception of ageing, while PL is reverse.

**Frailty** was assessed by the FRAIL scale, which included 5 items (Fatigue, Resistance, Ambulation, Illness, and Loss of weight). The FRAIL scale was constructed based on consensus of a European, Canadian and American Geriatric Advisory Panel [47]. It was showed similar predictive accuracy to both the Fried’s Frailty Phenotype and Rockwood and Mitnitski’s Frailty Index [48, 49]. The FRAIL scale was being increasing used in Asia-pacific region [50], and showed a favourable validity in community-based older Chinese with below and above 75 years old [51]. The criteria defined frail as the presence of 3 or more of these 5 symptoms, the presence of 1 or 2 defined prefrail, and 0 corresponded to robust.

### Covariates

Age, gender, education, marital status, economic condition and residence status were chosen as the potential confounding variables. Educational level was generally categorized into 5 levels (illiteracy, primary school, junior high school, high school or equivalent, and college or above). Marital status was divided into married and unmarried (never married, widowed and divorced). Economic condition was assessed by the question: “*How do you think of your current income and daily expenses?*” and the responses were “*income lower than expenditure, income equal expenditure, and income higher than expenditure*”. Residence status was measured by a multiple-choice question: “*Who are you currently living with?* (*alone, spouse, parents, child/ren, grandchild/ren, others*)”; and it was divided into live alone, live with spouse (only spouse), and live with others.

### Statistical analysis

To examine the hypothesized model in Fig. 1, we used Mplus 8.3 [52] for windows to appraise the SEM with latent variables. In the first step, the Chi-square test was performed to screen out the potential confounding variables by SPSS 22.0 for windows. Based on the results in Table 2, we selected all sociodemographic variables except for gender and economic condition as the covariates in the SEM. For analyses, we transformed the categorical variables into binary variables on the basis of merging the categories with similar percentages of frailty. For example, education was divided into primary school or below (0) and junior high school or above (1); residence status was divided into live alone (1) and not live alone (0). In addition, frailty was addressed into an ordered variable (robust = 1, prefrail = 2, frail = 3) according to the criteria. When a mixture of binary, ordered categorical, and continuous variables are included in SEM, analyses are usually based on polychoric/polyserial correlations [53].

**Table 2:** Univariate analysis of frailty status between different characteristics

Characteristics	Robust (%)	Prefrail (%)	Frail (%)	χ^2^/ F	*P* value
Gender				2.308	0.315
Male	106 (46.5)	93 (40.8)	29 (12.7)		
Female	166 (41.3)	170 (42.3)	66 (16.4)		
Age (years, mean ± SD)	72.49 ± 8.22	73.91 ± 8.25	79.85 ± 7.81	28.880	< 0.001
Age group				57.800	< 0.001
60–69 years	113 (51.6)	93 (42.5)	13 (5.9)		
70–79 years	93 (45.4)	92 (44.9)	20 (9.8)		
≥ 80 years	66 (32.0)	78 (37.9)	62 (30.1)		
Education				31.274	< 0.001
Illiteracy	18 (35.3)	16 (31.4)	17 (33.3)		
Primary school	24 (32.0)	31 (41.3)	20 (26.7)		
Junior high school	101 (44.1)	99 (43.2)	29 (12.7)		
High school or equivalent	91 (50.8)	72 (40.2)	16 (8.9)		
College or above	38 (39.6)	45 (46.9)	13 (13.5)		
Marital status				9.633	0.008
Married	214 (46.7)	183 (40.0)	61 (13.3)		
Unmarried	58 (33.7)	80 (46.5)	34 (19.8)		
Economic condition				9.485	0.050
Income lower than expenditure	27 (36.0)	31 (41.3)	17 (22.7)		
Income equal expenditure	137 (40.4)	153 (45.1)	49 (14.5)		
Income higher than expenditure	108 (50.0)	79 (36.6)	29 (13.4)		
Residence status				16.105	0.003
Live alone	38 (30.9)	54 (43.9)	31 (25.2)		
Live with spouse	156 (46.3)	137 (40.7)	44 (13.1)		
Live with others	78 (45.9)	72 (42.4)	20 (11.8)		

*SD* Standard Deviation

In the second step, we assembled the modified measurement models and the structural equations simultaneously to establish the proposed SEM. We employed the mean- and variance-adjusted weighted least squares (WLSMV) as the method of estimation because of the analyses included categorical endogenous variables, and the link was probit in current model. To improve model fit, we freed covariances between error terms based on their modification indices (M.I.) during the estimation process. There has been no universal rule as to which model fit indices should be chosen, therefore, the most common indices and acceptable reference values included the magnitude of χ^2^ divided by its degrees of freedom (χ^2^/df < 3), CFI (<.90), TLI (<.90) and RMSEA (<.08) were reported in our study [52]. There was no necessary to use any imputation method because of no missing cases in the sample. A *p* < .05 was considered statistically significant for all analyses.

## Results

Table 1 shows the descriptive statistics for sociodemographic variables, attitude to ageing and frailty status. The prevalence of frailty was 15.1%, about two halves of the rest were prefrail and robust elders. We found significant differences in sociodemographic characteristics among the three groups of frailty status and the results were presented in Table 2. Specifically, the risk of frailty increased with age, and the prevalence of frailty was more likely to be reported by those whose education were below junior high school, those who were unmarried (never married, divorced and widowed), and those who lived alone.

Table 3 provides summary compositions of EA and AS, and the results of their internal consistency and CFA. It is necessary to note the important finding. The model fit statistics indicated that the measurement of EA and AS were both reliable and valid in terms of the internal consistency and construct validity in current study. The Cronbach’s alpha of each sub/scale were greater than .80, and the model fit indices of CFA indicated good model fit of these two measurement models after linking the covariant residuals by double arrows according to their M.I.. For instance, ea1 and ea5 (*r* = .312), ea2 and ea6 (*r* = .436), ea4 and ea8 (*r* = .298), as3 and as4 (*r* = .492), the correlation demonstrated the co-variation between those items. The RMSEA estimate (.024) for measurement model of AS was lower than .05, and the values of CFI and TLI were higher than .95, suggesting good fit of the model. While the RMSEA estimate (.054) for measurement model of EA was lower than .08 also suggesting adequate fit of the model.

**Table 3:** Determination of internal consistency and confirmatory factor analysis (CFA) results for measurement models of experiences of ageism (EA) and age stereotypes (AS)

Latent construct		Manifest variable	Question	Positive (%)/ Mean (SD)	Internal consistency	CFA
					Cronbach’s Alpha	Factor loading
EA ^a^	Witnessed age stereotypes (E1)	ea1	Too old to be	243 (38.6)	0.857	0.784^***^
		ea2	Too old to do	222 (35.2)		0.853^***^
		ea3	Incompetence	205 (32.5)		0.817^***^
		ea4	Memory loss	281 (44.6)		0.649^***^
	Encountered age stereotypes (E2)	ea5	Too old to be	157 (24.9)	0.858	0.812^***^
		ea6	Too old to do	145 (23.0)		0.849^***^
		ea7	Incompetence	116 (18.4)		0.816^***^
		ea8	Memory loss	124 (19.7)		0.633^***^
	Perceived ageism (E3)	ea9	Prejudice	155 (24.6)	0.884	0.869^***^
		ea10	Disregard	134 (21.3)		0.922^***^
		ea11	Maltreat	93 (14.8)		0.760^***^
AS ^b^		as1	Too old to be (1–5)	2.52 (1.07)	0.832	0.858^***^
		as2	Too old to do (1–5)	2.56 (1.08)		0.898^***^
		as3	Incompetence (1–5)	2.92 (1.21)		0.657^***^
		as4	Memory loss (1–5)	3.22 (1.28)		0.464^***^

*SD* Standard Deviation

^***^ *p* < .001

^a^ Model fit index of CFA for the EA: χ^2^/df = 2.839, CFI = 0.982, TLI = 0.975 and RMSEA = 0.054

^b^ Model fit index of CFA for the AS: χ^2^/df = 1.373, CFI = 1.000, TLI = 0.998 and RMSEA = 0.024

**Fig. 2:**
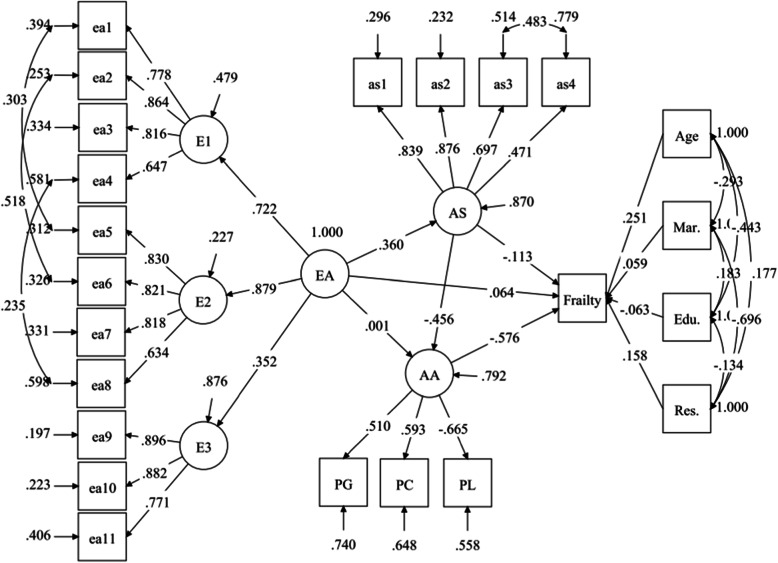
Direct and indirect effects of EA on frailty status with all standardized path coefficients. Note: E1 = Witnessed age stereotype, E2 = Encountered age stereotype, E3 = Perceived ageism, EA = Experience of Ageism, AS = Age Stereotype, AA = Attitude to Aging, PG = Psychological Growth, PC=Physical Change, PL = Psychosocial Loss, Mar. = Married, Edu. = Education, Res. = Residence; The goodness-of fit indices of the modified SEM were adequate: χ^2^/df = 2.329, CFI = 0.934, TLI = 0.921 and RMSEA = 0.046

For testing the hypothesized model, the proposed SEM was established and modified. Figure 2 shows the standardized solution for the proposed model that adjusted for age, education, marital status and living condition. The coefficients near to the single headed arrows are standardized regression weights (betas, β) and to the double headed arrows are standardized correlations, which both could be interpreted as correlations. This final model showed adequate goodness-of-fit indices: χ^2^/df = 2.329, CFI = .934, TLI = .921 and RMSEA = .046. The R^2^ of AS, AA and Frailty were .130, .208, .399, respectively, and several important findings were summarized as follows.

**Fig. 3:**
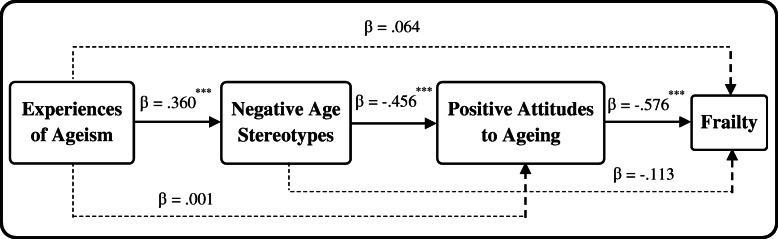
Conceptual and analytic model of ageism effects on frailty. The numbers nearby single headed arrows were direct effects of the paths in the structural equation model (^***^p < .001)

In the model, EA was constructed as a second-order-factor latent model by its three dimensions of witnessed AS, encountered AS and perceived ageism with the factor loadings of .72, .88 and .35, respectively. The summary of standardized direct and indirect effects of EA, AS and AA on Frailty were showed in Table 4. And the conceptual and analytic model of relationship between ageism and frailty was showed in Fig. 3. AA had a significant direct effect on Frailty (β = −.576, *p* < .001), it was to say, older people with a higher AA score was significantly correlated with lower probability of frailty. However, the direct effect of EA and AS on Frailty were not significant (β = .064, *p* = .286; β = −.113, *p* = .054, respectively). EA had a significant direct effect on AS (β = .360, *p* < .001) and AS had a significant direct effect on AA (β = −.456, *p* < .001). Overall, EA had a significant indirect effect on frailty only through the distal mediation of AS and AA (EA → AS→AA→Frailty: β’ = .360*-.456*-.576 = .095, *p* < .001) rather than them alone (EA → AS→Frailty: β’ = .360*-.113 = −.041, *p* = .071; EA → AA→Frailty: β’ = 0.001*-.576 = −.001, *p* = .985). EA had a significant indirect effect (β’ = .360*-.456 = −.164, *p* < .001) on AA through the full mediating effect of AS, and AS also had a significant indirect effect (β’ = −.456*-.576 = .263, *p* < .001) on Frailty through the full mediating effect of AA.

**Table 4:** Summary of standardized direct and indirect effects ^a^ of EA, AS and AA on Frailty status

Dependent variable	Independent variable (path)	Std. Est.	S.E.	Est./S.E.	*P* value	*R*^2^
Frailty	EA	0.117	0.054	2.187	0.029	0.399
	EA → Frailty	0.064	0.060	1.067	0.286	
	EA → AS → Frailty	−0.041	0.023	−1.802	0.071	
	EA → AA → Frailty	−0.001	0.035	−0.018	0.985	
	EA → AS → AA → Frailty	0.095	0.020	4.639	***	
	AS	0.149	0.053	2.842	0.004	
	AS → Frailty	−0.113	0.059	−1.924	0.054	
	AS → AA → Frailty	0.263	0.042	6.228	***	
	AA	−0.576	0.056	−10.213	***	
	Age	0.251	0.048	5.198	***	
	Married	0.059	0.062	0.959	0.338	
	Education	−0.063	0.047	−1.347	0.178	
	Residence	0.158	0.058	2.713	0.007	
AA	EA	−0.163	0.057	−2.863	0.004	0.208
	EA → AA	0.001	0.060	0.018	0.985	
	EA → AS → AA	−0.164	0.029	−5.614	***	
	AS	−0.456	0.050	−9.118	***	
AS	EA	0.360	0.051	7.067	***	0.130

***p < .001

## Discussion

Basically, the hypothesized model was supported by the results, which showed that EA influenced frailty through a full mediation path of AS and AA. It should be noting that the direct effect of ageism on frailty was not significant in current study. Even though the total effect of EA on frailty was not strong, this provided empirical evidence to the stress process model (SPM) [54], which demonstrated discrimination as a pressure source could influence health through the indirect path. To our knowledge, there has been no study to explain the relationship between ageism and frailty yet, while most of previous studies indicated the negative influence of ageism on mental health and self-rated health [6–8]. What’s more, the findings of this study were helpful to address a lack of understanding about the mechanism of ageism on frailty.

Using the SEM approach, we extended and integrated the correlations among EA, AS, AA and frailty. Previous experimental research indicated that AS can influence older people’s physical and mental performance [27, 37, 38]. Even though in current model, the direct effect of AS on frailty was not significant, the mediator of AA may link the correlation between AS and frailty. This was similar to the result of a previous experimental study that also showed a full mediation effect of SPA between AS and physical functioning [10]. To some extent, AS is a kind of subconscious cognition towards ageing or older people, and AA is likely the embodiment of this subconscious. More positive AA or SPA predicted better physical outcomes including frailty in previous community-based longitudinal studies [4, 41, 55]. Positive aspect of AS may improve positive AA and negative aspect of AS may enhance negative AA, which can have beneficial or detrimental health effects respectively.

Individuals internalize the AS across the life span, it was not always harmful to health until the negative aspect of AS was activated. As the result showed that EA had an indirect effect on AA by the mediator of AS; in other words, it demonstrated that more negative EA stimulated more negative AS, and more negative AS enhanced more negative AA. This matched the stereotype embodiment theory (SET) proposed by Becca Levy [16] who considered that individuals who were more frequently exposed to stereotypes are more likely to embody such stereotypes. Not only that, an experimental research previously has showed that implicit positive AS intervention can activate positive AS, which can enhance positive self-perceptions of ageing, and then improved physical functioning [10]. Although we explained this similar path from the negative perspective of AS, this also provided empirical evidence to the SET.

In sum, this study demonstrated a mechanism from ageism to frailty and provided an easier understanding for the influence of unconscious age-based stereotypes on actual health, although the effect of ageism on frailty was not strong. Furthermore, in this study, the definition of EA was expanded to a broader one, which not only included explicit ageism, but also experiences of implicit negative AS. We highlighted that these experiences of implicit negative AS should be identified and intervened within campaign to combat ageism because of overwhelming evidence indicating their negative health influence [11]. The determinants of frailty included a variety of physiological changes and/or diseases associated with ageing [50], and several psychological, social and environmental factors in previous studies [56–59]. However, we also emphasized that such a common but overlooked factor of ageism should be taken seriously in the process of frailty.

Some limitations are worth noting here. Firstly, this is a cross-sectional study and the direction of causation should not be entirely inferred from the proposed model, even though the SEM is recursive in nature. More future research should collect longitudinal data or experimental data to clarify the causality among these factors in the proposed framework. Secondly, the measures in this study are not exhaustive for the hypothesized model; especially, the assessments of EA and AS were not comprehensive enough because of their only negative valence and finite dimensions, and we did not examine the psychometric properties of the FRAIL scale. In future studies, psychometric and more valid instruments related to EA and AS should be used in the model, as well as tests related to different parts also could be considered in the proposed framework, such as using Fried’s phenotype criteria to assess frailty. Lastly, a convenient sampling method may lead to potential selection bias, which should be well controlled in future studies.

## Conclusion

These findings demonstrated a mechanism from ageism to frailty, and it provided an empirical evidence to the SET. Furthermore, this study expended a broader scope of EA, which included not only experiences of prejudice and discrimination because of age, but also experiences of negative age-based stereotyping. We highlighted the potential threat of negative AS on health. Ageism and frailty are both great challenges for the process of healthy ageing, and effective measures should be taken.

## Supplementary information

**Additional file 1.**

## Data Availability

The data applied and analyzed in the current study are available from the corresponding author upon reasonable request.

## Abbreviations

AA: Attitudes to ageing
AAQ: Attitudes to ageing questionnaire
AS: Age stereotypes
CFA: Confirmatory factor analysis
CFI: Comparative Fit Index
EA: Experiences of ageism
EFA: Exploratory factor analysis
FRAIL: Fatigue, Resistance, Ambulation, Illnesses, Loss of weight
GFI: Goodness of Fit Index
MI: Modification indices
PC: Physical change
PG: Psychological growth
PL: Psychosocial loss
RMSEA: Root Mean Square Error of Approximation
SD: Standard Deviation
SEM: Structural equation model
SET: Stereotype Embodyment Theory
SPA: Self-perception of ageing
SPM: Stress process model
TLI: Tucker-Lewis Index
