# Supplementary material for: How does ageism influence frailty? A preliminary study using a structural equation model
Source: BMC Geriatr. 2020 Oct 26;20:422. doi: 10.1186/s12877-020-01749-8 (PMC7586685; doi:10.1186/s12877-020-01749-8)
Supplement: Supplementary file 1 — Additional file 1. [file 12877_2020_1749_MOESM1_ESM.docx]

1. **Exploratory Factor Analysis (EFA) for Experiences of ageism (EA)**

| **KMO and Bartlett's Test** | | |
| --- | --- | --- |
| Kaiser-Meyer-Olkin Measure of Sampling Adequacy. | | .823 |
| Bartlett's Test of Sphericity | Approx. Chi-Square | 3996.414 |
|  | df | 55 |
|  | Sig. | .000 |

| **Total Variance Explained** | | | | | | | | | |
| --- | --- | --- | --- | --- | --- | --- | --- | --- | --- |
| Factor | Initial Eigenvalues | | | Extraction Sums of Squared Loadings | | | Rotation Sums of Squared Loadings | | |
|  | Total | % of Variance | Cumulative % | Total | % of Variance | Cumulative % | Total | % of Variance | Cumulative % |
| 1 | 4.802 | 43.658 | 43.658 | 4.307 | 39.157 | 39.157 | 2.453 | 22.299 | 22.299 |
| 2 | 2.115 | 19.230 | 62.888 | 1.963 | 17.842 | 57.000 | 2.422 | 22.022 | 44.321 |
| 3 | 1.182 | 10.747 | 73.635 | .821 | 7.468 | 64.468 | 2.216 | 20.147 | 64.468 |
| 4 | .722 | 6.568 | 80.203 |  |  |  |  |  |  |
| 5 | .454 | 4.127 | 84.330 |  |  |  |  |  |  |
| 6 | .418 | 3.798 | 88.128 |  |  |  |  |  |  |
| 7 | .363 | 3.304 | 91.432 |  |  |  |  |  |  |
| 8 | .309 | 2.811 | 94.243 |  |  |  |  |  |  |
| 9 | .263 | 2.393 | 96.636 |  |  |  |  |  |  |
| 10 | .194 | 1.761 | 98.397 |  |  |  |  |  |  |
| 11 | .176 | 1.603 | 100.000 |  |  |  |  |  |  |
| Extraction Method: Maximum Likelihood. | | | | | | | | | |


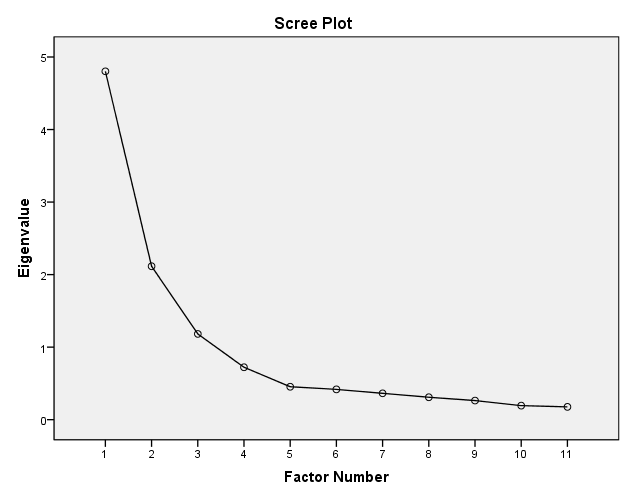


| **Rotated Factor Matrix^a^** | | | |
| --- | --- | --- | --- |
|  | Factor | | |
|  | 1 | 2 | 3 |
| witnessed1 | **.748** | .246 | .086 |
| witnessed2 | **.773** | .374 | .059 |
| witnessed3 | **.759** | .253 | .120 |
| witnessed4 | **.627** | .182 | .034 |
| encountered1 | .329 | **.730** | .129 |
| encountered2 | .318 | **.818** | .069 |
| encountered3 | .253 | **.740** | .173 |
| encountered4 | .183 | **.565** | .163 |
| perceived1 | .109 | .126 | **.850** |
| perceived2 | .076 | .122 | **.915** |
| perceived3 | .044 | .155 | **.743** |
| Extraction Method: Maximum Likelihood.  Rotation Method: Varimax with Kaiser Normalization. | | | |
| a. Rotation converged in 5 iterations. | | | |


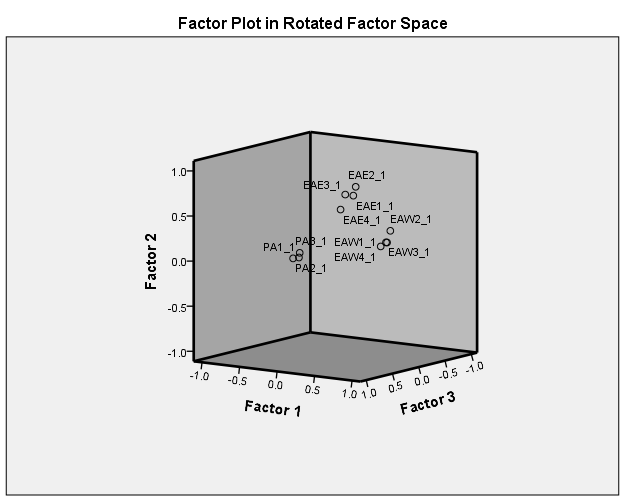


1. **Exploratory Factor Analysis (EFA) for Age Stereotypes (AS)**

| **KMO and Bartlett's Test** | | |
| --- | --- | --- |
| Kaiser-Meyer-Olkin Measure of Sampling Adequacy. | | .717 |
| Bartlett's Test of Sphericity | Approx. Chi-Square | 1187.635 |
|  | df | 6 |
|  | Sig. | .000 |

| **Total Variance Explained** | | | | | | |
| --- | --- | --- | --- | --- | --- | --- |
| Factor | Initial Eigenvalues | | | Extraction Sums of Squared Loadings | | |
|  | Total | % of Variance | Cumulative % | Total | % of Variance | Cumulative % |
| 1 | 2.694 | 67.345 | 67.345 | 2.267 | 56.682 | 56.682 |
| 2 | .753 | 18.832 | 86.177 |  |  |  |
| 3 | .325 | 8.117 | 94.294 |  |  |  |
| 4 | .228 | 5.706 | 100.000 |  |  |  |
| Extraction Method: Maximum Likelihood. | | | | | | |


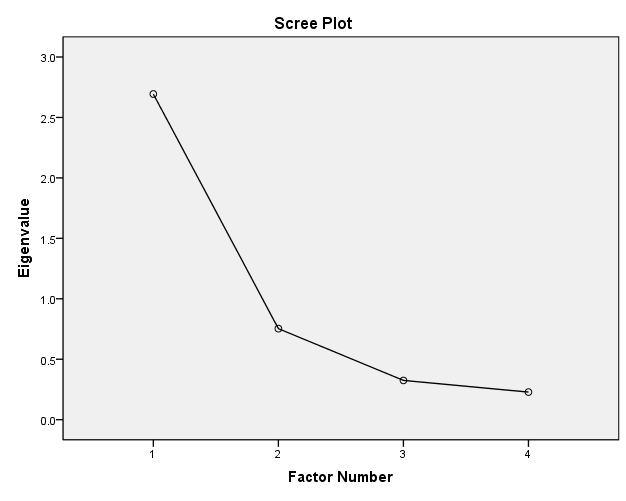


| **Factor Matrix^a^** | |
| --- | --- |
|  | Factor |
|  | 1 |
| AS1 | .849 |
| AS2 | .881 |
| AS3 | .699 |
| AS4 | .530 |
| Extraction Method: Maximum Likelihood. | |
| a. 1 factors extracted. 5 iterations required. | |
